# Retroperitoneal extra gastrointestinal stromal tumor: A case report^☆^

**DOI:** 10.1016/j.ijscr.2023.108442

**Published:** 2023-06-29

**Authors:** Ebaa Alabdallah, M.H.D. Moamen Al Mouallem, Basel Al-Ghotani, Nafiza Martini, Souheb Al-mahasna

**Affiliations:** Damascus University, Faculty of Medicine, Damascus, Syria; Stemosis for Scientific Research, Damascus, Syria

**Keywords:** Extra-gastrointestinal stromal tumor, Mesenchymal, Retroperitoneal tumor, Extra-intestinal tumor, Case report

## Abstract

**Introduction:**

Gastrointestinal stromal tumors (GISTs) are the most common mesenchymal tumors. Researchers do not know precisely what leads to GISTs, but genetic mutations play an important role. These mutations have no apparent cause. GISTs are usually asymptomatic tumors, although GI bleeding and weight loss can occur. CT is preferred for investigating potential GISTs.

**Case presentation:**

A 36-year-old unmarried Syrian female came to the hospital complaining of recurrent abdominal pain. CT revealed a large mass occupying a significant portion of the left hypochondrium and the lower part of the epigastrium. The tumor exceeded the median line to the right, pressing on the mesenteric vessels and the intestinal loops below. Immunohistochemistry results showed moderate positivity to CD117 and CD34, which were compatible with the diagnosis of GIST. The entire mass was excised. Physicians performed CT follow-ups every three months for 18 months, and no evidence of recurrence was observed.

**Discussion:**

Extragastrointestinal GISTs are a rare subtype of GISTs that occur outside the GI tract. GISTs previously used to be misdiagnosed as leiomyoma, leiomyosarcoma, leiomyoblastoma, and schwannoma. Treatment depends on surgery with adjuvant therapy tyrosine kinase inhibitors. Follow-up is recommended as the risk of recurrence is high.

**Conclusion:**

We recommend that GIST, as an extremely rare tumor, should be considered in the differential diagnoses of masses that occur in the extra-intestinal region. Usually, patients need surgery with lymph node resection. However, this was not needed in our case.

## Highlights

• Retroperitoneal GISTs are a rare subset of GISTs that can be confirmed by histology and immunophenotyping.
• Gastrointestinal stromal tumors (GISTs) can be detected incidentally or may present with symptoms. The most common symptoms include obscure or overt gastrointestinal bleeding and unexplained abdominal discomfort. Some symptoms occur less frequently, such as a palpable mass, perforation, or obstruction.
• Although GISTs can occur at any age, they are more prevalent in adults than in children. Certain factors increase the likelihood of developing GIST, such as inheriting a genetic mutation in the KIT gene, and to a lesser extent, the PDGFRA gene.
• Complete resection is the preferred treatment for primary low-risk GISTs, while resection with adjuvant imatinib 400 mg daily for 12 months is recommended for high-risk primary or metastatic tumors.

## 1 Introduction

Gastrointestinal stromal tumors (GISTs) are the most common mesenchymal tumors that mostly occur in the stomach and small intestines. They are generally defined as KIT (CD117)-positive tumors that have a distinctive set of histological features [1,2]. It overwhelmingly occurs in the stomach with a rate of 60 %, followed by the small intestine (jejunum and ileum) at 30 %, and the duodenum at 4–5 % [2]. Nevertheless, GISTs almost only occur in the stomach in children at a rate of <1 % [2]. Their general rate has been assessed as 10 to 20 for every million, including incidental minimal tumors [2]. It is more common in patients with neurofibromatosis-1, who are more predisposed than others to GISTs [2]. Researchers do not know precisely what leads to GISTs, but it occurs remarkably on a genetic level as genetic mutations are surprisingly not inherited. These mutations have no obvious reason (no known lifestyle-related or environmental risk factors) and are called acquired or sporadic [3]. The majority of those with GISTs have a mutation in the KIT oncogene (also known as CD117), and fewer people, about 5 % to 10 %, have a mutation in the PDGFRA gene [3]. The KIT protein drives the cell to grow and divide. Thus, if there is a mutation in this gene, there will be no control over the cell division of the interstitial cell of Cajal (ICCs) [3]. Furthermore, GISTs are usually asymptomatic tumors, although GI bleeding, intestinal obstruction, dysphagia (for tumors in the esophagus), and weight loss can occur [3]. GISTs have no predilection for sex. While the majority of the cases reported, about 90 % of them, were either children or older than 40 years old, with a median diagnosis age of 63 years [4,5]. Generally, computed tomography (CT) is preferred to investigate potential GISTs. An MRI image can provide further information about internal features and the detection of metastases [5]. Surgical resections with clear margins are the main treatment. But for some GIST tumors which are <2 cm and asymptomatic, doctors tend to monitor them carefully with an endoscopy on a regular basis, such as once or twice a year [6]. This manuscript has been reported in line with SCARE's 2020 Criteria [7].

## 2 Presentation

Herein, we present a case of a 36-year-old unmarried Syrian female who came to the hospital complaining of recurrent abdominal pain. She had lost a little weight and was in good general condition with no surgical, medical, or family history. Physical examination revealed a palpable mass around the umbilical region with mild tenderness.

Echography showed a well-defined oval mass with mixed content (a very turbid liquid tissue) behind the peritoneum. It extended from the level behind the stomach wall to the anterior of the pancreas with contact to the side of the pancreas behind the navel. It measures about 15, 17, and 20 cm, in addition to moderate to high turbid fluid in the abdomen and pelvis. Echo findings of this cystic mass could represent a hematoma, a pancreatic pseudocyst, or a developmental lesion. The right kidney appeared in a normal position, atrophic with irregular edges, and measured in its longitudinal diameter measures about 4 cm. Its corticomedullary differentiation was diminished, and it had several glossy coarse foci. The rest of the contents in the abdomen and pelvis were normal except for a small cystic cavity with clear content.

**Fig. 1:**
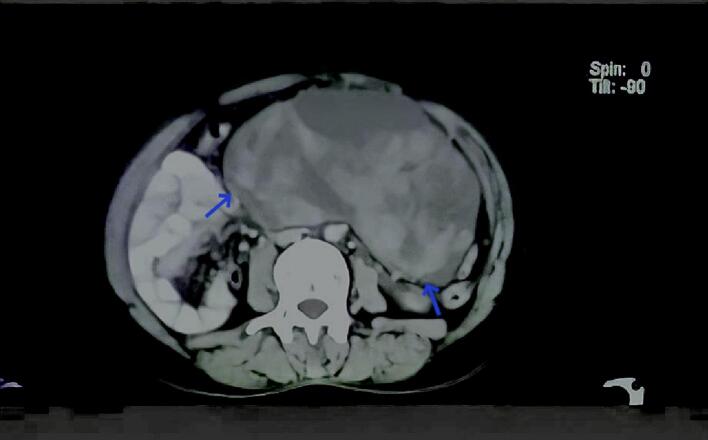
shows the large mass in the left hypochondrium, exceeding the median line to the right and pressing the mesenteric vessels.

Computed tomography (CT) of the abdomen and pelvis (Fig. 1) revealed a large mass measuring 20*16*10 cm, which occupied a large section of the left hypochondrium and the lower epigastrium, reaching from the lesser curvature of the stomach superiorly to the aditus of the greater pelvis inferiorly. The tumor exceeded the median line to the right, pressing the mesenteric vessels and the intestinal loops below. The mass was heterogeneous and necrotic with clear, non-perfused edges, which mostly corresponded to sarcoma. There was evidence of a medium amount of free fluid extending from the down edges of the mass to the minor pelvis. The CT also showed a regular homogenous accessory cyst with a thin wall on the left side that measured around 48 mm.

**Fig. 2:**
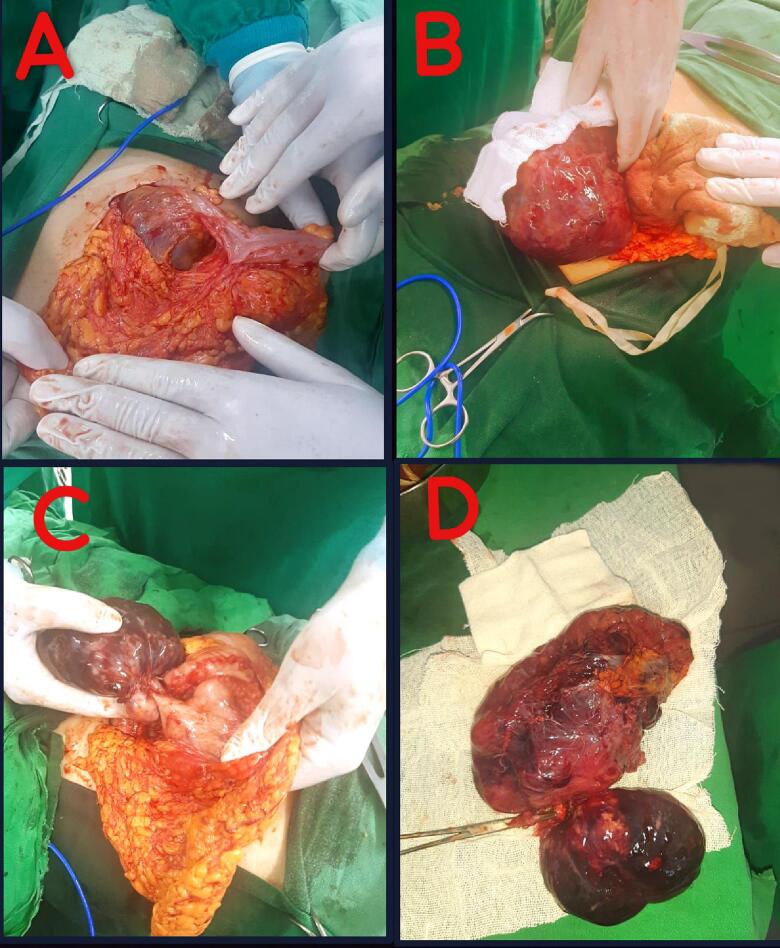
A, B, and C show the mass during surgery. D: Surgical specimen of resected GIST tumor.

In conclusion, the patient had a large mass of 20, 16, and 10 cm, an accessory cyst, and free fluid on CT. Thus, surgery was decided. A median surgical incision was made under general anesthesia, and the lesser sac was opened. A large mass consisting of two lobes/parts was found. One lobe had mixed content with a capsule surrounding it, which ruptured, and a dark serous fluid came out. The other lobe, which was a tissue mass, was loosely attached to the posterior wall of the stomach. The entire mass was excised without the presence of any vascular stalk of the mass, as well as no infiltrates in the stomach, pancreas, or mesenteric vessels (Fig. 2A, B, C, D). Following the surgery, the patient was put on an Imatinib plan for three years with follow-up.

**Fig. 3:**
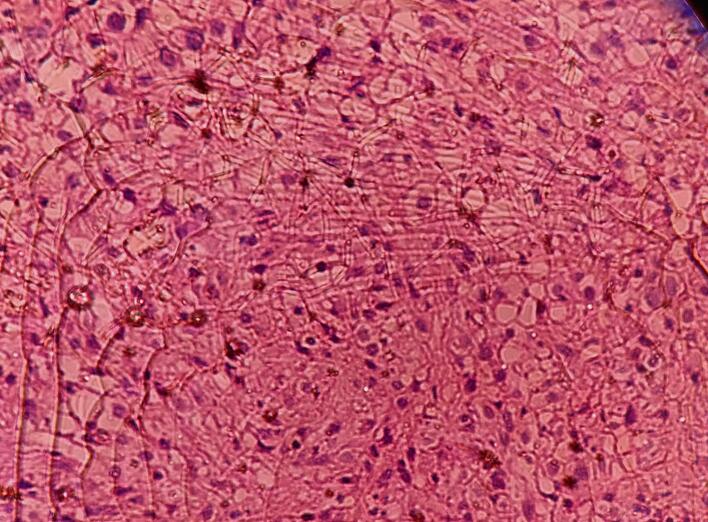
(H&E stain, x40): Histological examination shows predominant spindle cell morphology with cytoplasmic vacuolization, myxoid stroma.

After resection and on macroscopic examination, the tumor was well-demarcated and encapsulated. The cut surface had a homogeneous appearance of soft myxoid/edematous beige tissue. Also, the other mass that measured 8 cm had a similar cut surface and wide areas of hemorrhage. On the H&E level, the tumor showed predominant spindle cell morphology with cytoplasmic vacuolation, myxoid stroma, and vascular-like spaces, consistent with vascular neoplasm. The case summary according to the 2020 CAP protocol was as follows: the histologic type according to the WHO classification was suggestive of vascular sarcoma, the tumor mitotic rate > 5/ 5 mm^2 was that of a high grade with a high-risk assessment, necrosis also scored 1, differentiation scored 3, margins were uninvolved by sarcoma, Lymphovascular invasion was not identified, no lymph nodes were found, pathologic stage classification (pTNM, AJCC 8th Edition) was pT4 pNx. (Fig. 3) Immunohistochemistry resulted in diffused moderate positivity to CD117 and CD34, but negative to CD31 and S100. These results and H&E features were considered diagnostic of GIST.

The patient was discharged from the hospital the next day after the surgery. The patient underwent periodic follow-up physical examinations, in which the abdominal ultrasound and CT were performed every 3 months, and no evidence of recurrence was observed during the whole 18 months.

## 3 Discussion

GISTs had been misdiagnosed as until leiomyoma, leiomyosarcoma, leiomyoblastoma, and schwannoma. Mazur and Clark first described the concept of gastrointestinal stromal tumor (GIST) in 1983 [8]. In the past, GISTs were called gastrointestinal autonomic nerve tumors (GANTs) and were described as GI smooth muscle tumors [9,10]. Also, the most common mesenchymal tumors of the GI tract are GISTs [11,12]. GISTs arise from Cajal cells, also called interstitial cells of Cajal (ICCs). About 60 %–70 % of GISTs are found in the stomach, and about 25 %–30 % are in the small intestine, but they less frequently occur in the colorectum (5 %—15 %), duodenum (5 %), and esophagus (< 2 %). Extraintestinal GISTs are rare in <1 % of all GISTs. Moreover, we can find them in unexpected regions, such as the omentum, mesentery, gallbladder, pancreas, and retroperitoneum. Doctors believe that the size of the tumor, its location in the GI tract, the mitotic rate, and other factors may let the tumor spread and grow faster [3,13]. The five-year survival of GISTs is between 35 % and 65 %, depending on location, tumor size, and mitotic index [14]. According to the study, the principal event of GIST pathogenesis is the activation of Kit signal transduction, which leads to the productio676n of Kit protein (CD117) [11,12]. GISTs could occur from the distal half of the esophagus to the anorectal area. The incidence of GISTs ranges between 10 and 20 per million, which means that there are 3000 to 6000 new cases every year in the United States [15,16]. GISTs could be diagnosed incidentally or could be symptomatic tumors. The most common symptoms are ambiguous or visible GI bleeding and mysterious abdominal complaints. Some symptoms are less frequent, such as a palpable mass, perforation, or obstruction. Moreover, malignant GISTs could metastasize into abdominal soft tissues and liver, peripheral soft tissues, and rarely into bone (especially axial skeleton). Usually, 1 to 2 years after complete excision, metastases develop and could develop 5 to 15 years or more after primary surgery [17]. Nevertheless, GISTs are more common in adults (> 50) than children, although they can occur at any age [1]. Other factors make you more likely to develop GIST, such as inheriting a genetic mutation in the KIT gene and, to a lesser extent, the PDGFRA gene. In previous years, before KIT and PDGFRA gene tests became available, patients with familial GIST were misdiagnosed as neurofibromatosis because they had similar skin patches [18]. Gastrointestinal stromal tumors (GISTs) are mainly present in the tubular gastrointestinal tract. But, it is clear by histology and immunophenotypes that a subset of GIST is found outside the gastrointestinal tract. These tumors are known as extragastrointestinal GISTs or EGISTs [19,20]. EGISTs reported cases in the retroperitoneal location, and their data are sparse [19].

Screening tests for tumors like GISTs are not a good choice. Some GISTs are discovered early by chance, but many are discovered because of a person's symptoms. So, testing asymptomatic people is not recommended [21]. In GIST, CT scans help guide biopsy so that bleeding and the risk for metastasis are minimal [22]. However, new studies show no relevant risk of recurrence after pre-treatment biopsy [23]. Like CT, MRI helps in searching for recurrence and locating tumors in the abdomen, but CT is the usual choice for the last point [24]. GISTs are hard to differentiate in endoscopy as they are often in the submucosal region of the GI tract and appear as a submucosal bulge [22]. The biopsy is the way to a clear diagnosis, but because GISTs are often fragile tumors and bleed easily, some researchers think the risk of cancer spreading might increase [22].

The treatment for gastrointestinal stromal tumors (GISTs) is usually surgery if GISTs haven't spread with R0 resection as the objective of surgery [6,24]. Minimal complications after surgery, such as wound infection and chest infection may present and therefore require appropriate management [25]. The risk of recurrence after surgery is crucial, as further treatment depends on it [6]. In more detail, primary low-risk GISTs are treated with complete resection, high-risk primary or metastatic tumors with resection, and imatinib 400 mg daily for 12 months. If the tumor is unresectable, surgical resection follows neoadjuvant imatinib 400 mg daily [26].

In non-ruptured GISTs, the mitotic rate is the most important prognostic factor determining when adjuvant therapy is necessary. Tumor size, mitotic count, location, and tumor rupture have become known prognostic factors that can help predict recurrence, metastasis, and consideration for adjuvant therapy [27]. According to modified National Institutes of Health (NIH) consensus criteria, a high-risk GIST is >5 cm, has a mitotic count >5/50 HPF, or the tumor has ruptured. A low-risk GIST is 2.1–5 cm and has a mitotic count <5/50 HPF [23]. Here because the tumor is >10 cm and ruptured during surgery, it is considered a high-risk tumor; thus, surgical resection is the treatment of choice, and adjuvant therapy following surgery is necessary.

Adjuvant therapy for GISTs depends on tyrosine kinase inhibitors (TKI). Three approved agents are sunitinib (Sutent), imatinib (Gleevec), and ponatinib [26]. Imatinib is a TKI that blocks signal transduction after binding to the ATP binding sites on CD117 and PDGFRA. GISTs that are CD117 and PDGFRA positive could benefit from this therapy [26]. Exon 9 mutations and wild-type GISTs (no CD117 or PDGFRA mutations) are treated with sunitinib. Also, patients with exon 13 or 14 mutations benefit from sunitinib, while exon 17 mutations benefit from ponatinib, which is effective in treating resistant GISTs, although further investigation for adverse effects is needed [26]. Thus, imatinib is the drug of choice for therapy in our case. In the high-risk category, patients are recommended for follow-up with serial CT scans in addition to surgery and targeted therapy, approximately 10 years from imatinib initiation [27]. Our patient was on follow-up for 18 months, and CT showed no evidence of recurrence.

EGISTs may have a worse prognosis than conventional GISTs with high mitotic indices, large size, and distant metastasis. Conventional GIST patients compared with EGIST patients have no statistically significant difference in recurrence-free survival [28].

## 4 Conclusion

This study provided an exceptional posture for GIST as an abdominal retroperitoneal mass. Usually, the patient must be followed by surgery with the resection of the lymph nodes, which none of them were in our case. We recommend that GIST, as an extremely rare tumor, should be considered in the differential diagnoses of masses that occur in the extra-intestinal region.

## Abbreviations

GISTs: Gastrointestinal stromal tumors
EGISTs: Extra-gastrointestinal stromal tumors
GANTs: Gastrointestinal autonomic nerve tumors

## Consent for publication

Written informed consent was obtained from the patient for the publication of this case report and any accompanying images and videos. A copy of the written consent is available for review by the editor of this journal upon request.

## Provenance and peer review

Not commissioned, externally peer-reviewed.

## Ethical approval

Ethical approval for this study was provided by the Ethical Committee of Damascus University, Damascus, Syria on 20 June 2022, With number 4369.

## Funding

Not applicable.

## Authors' contribution

EA is the first author who contributed to drafting, reviewing, editing, data collection, and bibliography. Mhd M.A contributed to drafting, reviewing, editing, and bibliography. BA contributed to drafting, reviewing, editing, and bibliography. NM is the corresponding author and also contributed to drafting, reviewing, and editing. SM is the supervisor and contributed to data collection and reviewing. All authors have read and approved the manuscript.

## Guarantor

Dr. Souheb Al-mahasna.

## Research registration number

This study is a case report, so we can't make the registration it as a trial.

## Conflict of interest statement

We declare no conflict of interest.

## Data Availability

Not applicable.
